# Defect Detection for Enhanced Traceability in Naval Construction

**DOI:** 10.3390/s25041077

**Published:** 2025-02-11

**Authors:** Paula Arcano-Bea, Manuel Rubiños, Agustín García-Fischer, Francisco Zayas-Gato, José Luis Calvo-Rolle, Esteban Jove

**Affiliations:** Department of Industrial Engineering, University of A Coruña, CTC, CITIC, 15403 Ferrol, Spain

**Keywords:** convolutional autoencoders (CAEs), anomaly detection, shipbuilding, quality control, unsupervised learning

## Abstract

The digitalization of shipbuilding processes has become an important trend in modern naval construction, enabling more efficient design, assembly, and maintenance operations. A key aspect of this digital transformation is traceability, which ensures that every component and step in the shipbuilding process can be accurately tracked and managed. Traceability is critical for quality assurance, safety, and operational efficiency, especially when it comes to identifying and addressing defects that may arise during construction. In this context, defect traceability plays a key role, enabling manufacturers to track the origin, type, and evolution of issues throughout the production process, which are fundamental for maintaining structural integrity and preventing failures. In this paper, we focus on the detection of defects in minor and simple pre-assemblies, which are among the smallest components that form the building blocks of ship assemblies. These components are essential to the larger shipbuilding process, yet their defects can propagate and lead to more significant issues in the overall assembly if left unaddressed. For that reason, we propose an intelligent approach to defect detection in minor and simple pre-assembly pieces by implementing unsupervised learning with convolutional autoencoders (CAEs). Specifically, we evaluate the performance of five different CAEs: BaseLineCAE, InceptionCAE, SkipCAE, ResNetCAE, and MVTecCAE, to detect overshooting defects in these components. Our methodology focuses on automated defect identification, providing a scalable and efficient solution to quality control in the shipbuilding process.

## 1. Introduction

The integration of digital technologies has deeply transformed the manufacturing industry, improving production processes and significantly increasing efficiency [1]. This digital transformation offers unique benefits compared to traditional methods, such as getting information out quickly and making efficient use of resources, which are essential for modern industrial competitiveness [2]. Furthermore, innovation has emerged as a key driver of economic growth and an essential tool for businesses aiming to secure a competitive edge in global markets. By fostering advances in technology and processes, digitalization not only drives industries forward but also contributes to wealth creation and broader economic development [3].

In the shipbuilding sector, these advancements are of significant importance. Shipyards face mounting pressure to improve the quality of their final products to maintain relevance in a highly competitive market [4]. High-quality outputs not only enhance customer satisfaction but also play a decisive role in securing future contracts. For shipowners, product quality translates directly into trust and reliability. As a result, robust and structured quality control processes have become indispensable in the shipbuilding industry, having significant economic implications [5].

Nowadays, the shipbuilding industry is undergoing a profound transformation driven by the adoption of digital technologies aimed at improving efficiency, quality, and traceability throughout the production process [6]. By enabling manufacturers to monitor and document each stage of production, traceability ensures that defects can be promptly identified, their root causes determined, and appropriate corrective actions taken. This is vital for maintaining the structural integrity of vessels and avoiding costly failures during or after construction.

In today’s global shipbuilding market, modernization has become a critical priority for European shipyards to remain competitive, particularly in the face of growing competition from Asian shipyards [7]. To address these challenges, European shipyards, including those in Spain, often depend on a network of subcontractors that play an integral role in their production process [8]. This reliance highlights the necessity for these shipyards and their auxiliary partners to adopt digitalization tools and strategies. By integrating digital technologies into their operations, shipyards can facilitate collaboration with subcontractors, enhance production efficiency, and ensure better traceability and quality control. Such advancements are essential for maintaining competitiveness and fostering innovation and adaptability in an increasingly technology-driven industry.

One of the many challenges in achieving good quality control lies in the detection of defects in sub-assemblies. These components, which include minor and simple sub-assemblies, serve as the smallest building blocks of a larger ship block. Even minor defects in these parts can propagate through the assembly process, leading to significant quality issues in the final structure.

In recent years, machine learning and computer vision techniques have shown great potential by automating defect detection processes [9]. Among these, Convolutional Autoencoders (CAEs) have gained prominence for their ability to learn compact representations of data and detect anomalies in an unsupervised manner.

While several studies have focused on defect detection within the shipbuilding sector, most existing approaches rely on traditional or human-dependent inspection methods, with limited exploration of advanced machine learning techniques. For instance, research on traditional shipbuilding in Indonesia utilized logic tree analysis to identify potential defects but did not incorporate machine learning methods [10]. Similarly, studies have employed deep learning for detecting painting defects in shipyards [11], or machine learning algorithms for weld inspection [12], but none have explored the use of Convolutional Autoencoders (CAEs) for defect detection.

Recent advancements in deep learning have demonstrated the effectiveness of autoencoders in defect detection, particularly in improving feature extraction and anomaly identification. For example, deep autoencoder-based methods have been used to extract nonlinear features from thermographic data, improving defect visibility and detection accuracy [13]. Similarly, convolutional autoencoders combined with graph-based feature mapping have shown promise in reducing noise and highlighting defects in composite materials [14]. Additionally, a novel approach incorporating prior knowledge into convolutional autoencoders has shown strong generalization performance, particularly when dealing with small sample sizes and domain shifts [15]. These approaches highlight the potential of autoencoder architectures for unsupervised defect detection, making them a valuable tool for identifying anomalies in complex manufacturing processes.

While most research in the field of defect detection has focused on supervised learning methods, these approaches have significant limitations, particularly due to the reliance on labeled data [16]. Labeled data are often difficult to obtain, especially in industries like shipbuilding, where defects can vary in size, type, and location. In contrast, unsupervised learning methods, such as convolutional autoencoders, offer a compelling alternative. By training on defect-free data, autoencoders can identify anomalies without the need for labeled examples [16].

There are multiple unsupervised learning techniques for defect detection, Convolutional Autoencoders (CAEs) offer unique advantages that make them particularly suitable for this task. Unlike traditional unsupervised methods, such as Principal Component Analysis (PCA), which rely on linear transformations, CAEs capture complex, high-dimensional patterns through nonlinear feature extraction, making them more effective for identifying subtle defects [17]. Additionally, CAEs learn hierarchical representations, allowing them to differentiate between normal and anomalous patterns with greater precision. This has been demonstrated in semiconductor manufacturing, where CAEs outperformed classical feature extraction techniques in handling intricate, structured data [18]. Furthermore, CAEs have been successfully applied in image compression, proving their ability to efficiently encode and reconstruct detailed information [19].

By training CAEs on non-defective samples, they can effectively identify deviations in defective samples based on reconstruction errors [20]. This capability is particularly useful in scenarios where labeled defective samples are scarce or where the exact nature of defects is unpredictable.

This study focuses on the use of Convolutional Autoencoders (CAEs) to detect defects in 3D printed representations of minor and simple sub-assemblies, whose blueprints were provided by a company acting as a subcontractor to a shipyard. To achieve this, we will evaluate the performance of five distinct CAE architectures: BaseLineCAE, InceptionCAE, SkipCAE, ResNetCAE, and MVTecCAE, analyzing their ability to differentiate between defective and non-defective pieces.

The defect we want to identify is an overshoot during the cutting process. A challenging issue due to its unpredictable occurrence in terms of location and severity. To address this, we employ an unsupervised learning approach, training the models exclusively on non-defective pieces. This strategy allows the detection of anomalies without prior knowledge of the defect’s specific characteristics or location.

Model performance will be assessed using two complementary metrics: Mean Square Error (MSE), which measures reconstruction accuracy, and Structural Similarity Index Measure (SSIM), which evaluates the perceptual similarity between the reconstructed and original images. Together, these methods aim to ensure robust and reliable defect detection.

The structure of this document is as follows: it begins with an introduction outlining the background and motivation for the study. This is followed by an explanation of the case study. Next, the approach and methodologies used for implementation are detailed. The subsequent sections describe the experiments conducted, present the results, and provide their analysis. Finally, the document concludes with a discussion of the findings and suggestions for future work.

## 2. Case of Study

For this study, we used real-world data provided by a company that operates as a subcontractor to a shipyard. This company specializes in metal manufacturing and supplies a wide range of components that are essential to the shipbuilding process. Given the complexity of shipbuilding, strict quality control of each component is essential to ensure that they meet the shipyard’s strict standards.

One of the critical stages of production is the metal-cutting process, which requires special attention. As mentioned in the introduction, cutting machines occasionally experience overshoot problems and deviate from their intended paths. When these deviations exceed acceptable thresholds, the affected parts are classified as defective, which highlights the importance of closely monitoring this process.

To contribute to this supervision process, we propose a system that identifies defective pieces through image analysis. Since the specific location or frequency of the overshoot problem within the pieces is unknown, we employ an unsupervised learning approach. Our methodology involves training the model exclusively on images of non-defective parts. In particular, we use convolutional autoencoders (CAEs), which are well-suited for this purpose because they reconstruct the original input image.

CAEs are excellent for unsupervised learning because the model learns to reproduce the features of non-defective pieces during training. When presented with an image of a defective part during testing, the model struggles to reconstruct it accurately, resulting in a higher reconstruction error. This discrepancy serves as a reliable indicator of defects.

The pieces that we will focus on in this study are minor and simple sub-assemblies, that, although they are some of the smallest components in the shipbuilding industry, are still significant in size. Even the smallest of these components measure nearly one meter in length and weigh over 11 kg.

Due to the significant dimensions of the components being studied, we decided to use 3D printing technology to create scaled-down versions of these parts, making them more manageable for testing and experimentation. This approach allowed us to simulate and study the problem under controlled conditions without the logistical challenges of handling the original, larger components.

We created 3D-printed replicas of both non-defective and defective parts. For the defective parts, we intentionally replicated the overshoot problem observed in the cutting process to ensure that the deviations accurately represented the real-world problem. This included varying the magnitude and shape of the deviations to capture a range of defect scenarios, thereby providing a more realistic dataset for testing our model.

**Figure 1:**
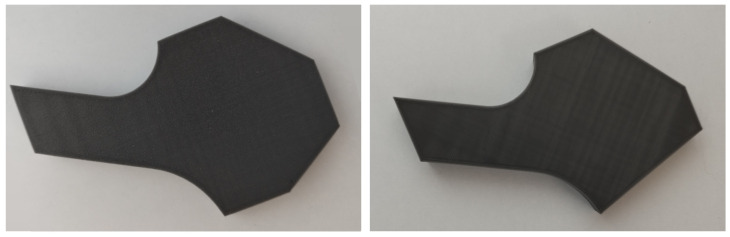
Example of two 3D printed pieces, a non-defective (**left** image) and a defective (**right** image).

In Figure 1, we have an example of two 3D-printed pieces: one non-defective (left image) and one defective (right image).

**Figure 2:**
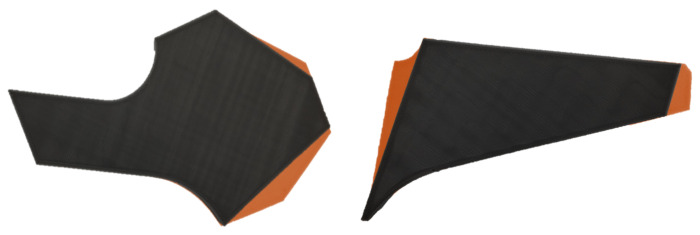
Defective Piece with Highlighted Defects in Orange.

To make it easier to identify the defects in the two images (Figure 1), Figure 2 is presented, where we have two different defective pieces; in this case, the defective piece is shown in gray and the contour of the non-defective piece is highlighted in orange. This contrast allows us to clearly see that the orange areas represent the defects in the piece.

## 3. Approach

**Figure 3:**
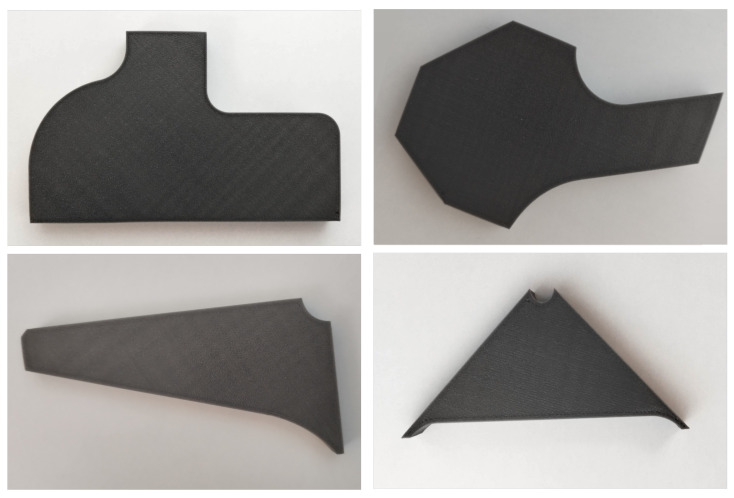
Pictures of the four different pieces.

To begin our study, we selected four different pieces (Figure 3) for analysis: two simple sub-assemblies and two minor sub-assemblies. Using 3D printing technology, we recreated these pieces as defect-free replicas and also created versions with overshooting defects to simulate the real-world problems we were aiming to identify.

The next step was to create our image dataset. To conduct this, we captured (with an Intel RealSense D455 Depth Camera) a large number of RGB images for both defective and non-defective versions of each piece, collecting over a hundred images per piece type. Given the relatively small size of the dataset, we applied extensive data augmentation techniques, including horizontal and vertical flipping, as well as brightness and saturation adjustments. These techniques were implemented not only to mitigate overfitting but also to enable the model to adapt to the complex variations in the context of industrial processes. As highlighted by previous research [21], data augmentation techniques, such as those used in this study, have proven effective in overcoming the challenges of limited datasets and improving the generalization of deep learning models in multiple applications. By augmenting the dataset in this way, we aimed to simulate real-world conditions and extend the model’s ability to detect defects to a wider range of potential scenarios.

Once the dataset was prepared, we divided it into training and test subsets. For the non-defective parts, we assigned 80% of the images to training and the remaining 20% to testing. The testing dataset also included images of defective parts to evaluate the model’s ability to detect anomalies. For the validation, we split 80% of the images of non-defective pieces into 80% training and 20% validation.

In this study, we will evaluate the performance of five different convolutional autoencoders: the BaseLineCAE, InceptionCAE, SkipCAE, ResNetCAE, and MVTecCAE. The goal of this comparison is to identify the most effective architecture for detecting overshoot errors in our dataset.

**Figure 4:**
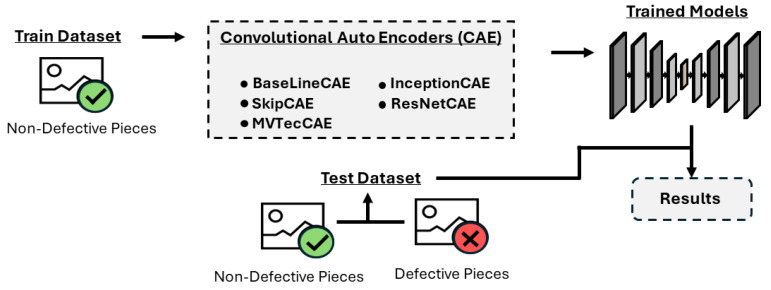
Visual representation of the approach.

In Figure 4 we have a graphical representation of our approach.

## 4. Methods and Materials

In this section, we will explain all the methods that were used in this study.

### 4.1. Physical Device

To capture the RGB images needed to create our dataset, we used the Intel RealSense D455 Depth Camera, a device known for its imaging capabilities. The camera is equipped with an RGB sensor that provides a resolution of 1280 × 800 pixels and captures images at a consistent frame rate of 30 frames per second. Its global shutter technology ensures that images are free of motion blur, making it ideal for capturing accurate visual data even when there is movement in the scene. The camera’s sensor has a wide field of view (FOV) of 90 degrees horizontally and 65 degrees vertically, allowing a large scene to be captured in a single image. This wide-angle capability is advantageous when imaging larger objects or multiple components in a single image. Although the D455 has depth sensing capabilities and a depth range of up to 6 m, this study did not employ the depth functionality. Instead, we focused completely on the camera’s high-quality RGB output. This decision allowed us to take full advantage of the D455’s reliable and detailed color imaging capabilities, ensuring the accuracy and clarity needed to train and test our defect detection models.

Additionally, the Intel RealSense D455 incorporates a Bosch BMI055 inertial measurement unit (IMU), which measures the camera’s acceleration and angular velocity and is designed for environmental adaptability and is capable of maintaining performance in both indoor and outdoor locations.

**Figure 5:**
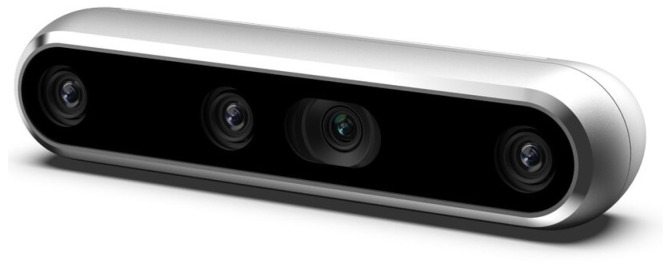
Intel RealSense D455 Depth Camera.

The Intel RealSense D455 is supported by RealSense Viewer software, an intuitive tool available for download online. This user-friendly application gives users real-time control over the camera’s functionality. The software allows users to view and manage live streams, including RGB and infrared data, while adjusting a wide range of camera parameters, such as resolution, frame rate, exposure and gain. The RealSense Viewer provides a seamless user experience, allowing for precise adjustments and optimal image capture settings tailored to specific requirements. Figure 5 presents an image of the Intel RealSense D455 camera.

Using the RealSense Viewer software, we adjusted multiple settings to ensure optimal image quality in our context. These adjustments included configuring the RGB resolution to the maximum (1280 × 800 pixels) and manually fine-tuning parameters, such as exposure and gain, to adapt to the lighting conditions of the environment. Additionally, saturation settings were modified to improve color accuracy and consistency across the dataset. These adjustments helped ensure that the RGB images met the required standards for our analysis.

### 4.2. Convolutional Autoencoders

**Figure 6:**
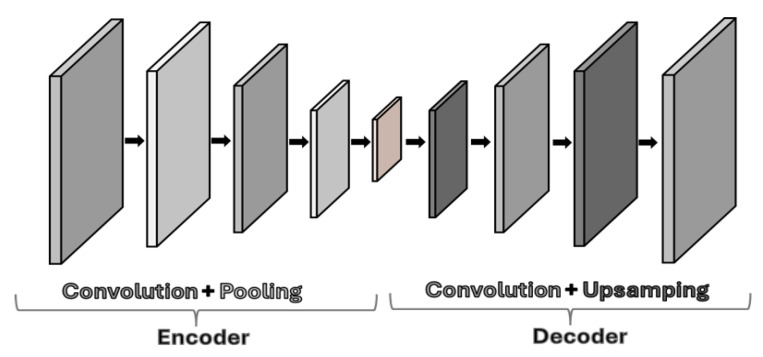
Example of the layers of a convolutional autoencoder.

Convolutional Autoencoders (CAEs) are deep neural networks designed to handle high-dimensional and complex data efficiently. They achieve this by using convolutional layers that capture local features in the input data. This local feature extraction allows CAEs to reduce dimensionality without losing critical information, making them particularly effective for applications that require preserving structural and spatial detail. This makes CAEs particularly effective for tasks, such as feature extraction, image reconstruction, and data compression because they achieve compression without a significant loss of fidelity to the underlying data structure [22]. In essence, CAEs operate as a subclass of convolutional neural networks (CNNs). The CAE architecture involves an encoding and decoding process using convolutional operations [23]. In the encoding phase, convolutional layers extract local features and pooling layers downsample the data to reduce its dimensionality. The decoding phase reverses this process using deconvolution and upscaling to reconstruct the input from its compressed representation [24]. In Figure 6, we have a visual representation of the most commonly used layers of a CAE.

The utility of CAEs for image defect detection is based on their ability to model normal data representations through reconstruction. During the training phase, CAEs operate in an unsupervised learning framework [25], where they are exposed only to images of defect-free components. This allows the network to autonomously learn an implicit representation of normality without the need for labeled data. When exposed to images of defective parts during the inference process, the network struggles to reconstruct anomalies that deviate from the learned normal patterns. This discrepancy manifests as higher reconstruction errors in regions corresponding to the defects.

By thresholding the reconstruction error, CAEs can effectively identify and localize defects in images. Also, the convolutional nature of the network ensures that spatial irregularities, such as surface cracks, deformations, or missing features, are easily detected, even in high-resolution images. This makes CAEs a powerful tool for automated visual inspection in manufacturing, where early and accurate defect detection is critical to ensure quality.

There are several commonly used CAEs, most of which are based on existing CNN architectures. Some of these Convolutional Autoencoders are as follows:

**BaseLineCAE**, whose architecture is based on a classic convolutional autoencoder (CAE) design with a symmetric layer structure. This architecture employs LeNet-type CNN architectures [26], which consist of an encoder and a decoder, where the encoder reduces the dimensions of the original image through convolutional layers followed by leaky ReLU activations and 2 × 2 max-pooling. On the decoder side, transposed convolutional layers and up-scaling are used to reconstruct the image from its compressed representation.

Mathematically, the encoder and decoder processes for BaselineCAE are described as follows:

Encoder:

$$h=\sigma (W_{\mathrm{enc}}*x+b_{\mathrm{enc}})$$

where

- *x* is the input image.
- $W_{\mathrm{enc}}$ and $b_{\mathrm{enc}}$ are the weights and biases of the encoder.
- ∗ denotes the convolution operation.
- *h* is the encoded representation.
- σ is the activation function (e.g., ReLU).

Decoder:

$$x^{\prime }=\sigma \left(W_{\mathrm{dec}}*h+b_{\mathrm{dec}}\right)$$

where

- $x^{\prime }$ is the reconstructed output.
- $W_{\mathrm{dec}}$ and $b_{\mathrm{dec}}$ are the weights and biases of the decoder.
- σ is the activation function.

**InceptionCAE**, is a variant of convolutional autoencoders that adopts the principles of the Inception network architecture [27]. This adaptation benefits from the power of the Inception architecture, which is known for its ability to efficiently capture multi-level features. The InceptionCAE integrates multiple convolutional filters with different kernel sizes into the encoder, allowing it to extract different features at different spatial resolutions. This multi-scale approach enhances the network’s ability to learn complex patterns and finer details in the data.

Mathematically, the encoder and decoder processes for InceptionCAE are described as follows:

Encoder: The encoder applies multiple convolutional filters of varying sizes in parallel:

$$h_{1}=\sigma \left(W_{1}*x+b_{1}\right)\;\left(1\;\times \;1\;\mathrm{filter}\right)$$

$$h_{2}=\sigma \left(W_{2}*x+b_{2}\right)\;\left(3\;\times \;3\;\mathrm{filter}\right)$$

$$h_{3}=\sigma \left(W_{3}*x+b_{3}\right)\;\left(5\;\times \;5\;\mathrm{filter}\right)$$

where

- *x* is the input image.
- $W_{1},W_{2},W_{3}$ are the weights of the filters with kernel sizes 1 × 1, 3 × 3, and 5 × 5, respectively.
- $b_{1},b_{2},b_{3}$ are the biases corresponding to each filter.
- ∗ represents the convolution operation.
- σ is the activation function (ReLU or LeakyReLU).
- $h_{1},h_{2},h_{3}$ are the feature maps resulting from each filter.

These outputs are then concatenated:

$$h_{\mathrm{inc}}=\mathrm{concat}\left(\sigma \left(W_{1}*x+b_{1}\right),\sigma \left(W_{2}*x+b_{2}\right),\ldots \right)$$

where

- concat is the concatenation operation that merges the outputs from the multiple convolutional layers with different kernel sizes $W_{1},W_{2},\ldots$.
- *x* is the input image.
- σ is the activation function (e.g., ReLU or LeakyReLU).
- $W_{1},W_{2},\ldots$ represent the convolution filters for each kernel size.
- $b_{1},b_{2},\ldots$ are the bias terms.

Decoder: The decoder reconstructs the image from the encoded features using upsampling and convolution operations:

$$x^{\prime }=\sigma \left(W_{\mathrm{dec}}*h_{\mathrm{inc}}+b_{\mathrm{dec}}\right)$$

where

- $x^{\prime }$ is the reconstructed output image,
- $W_{\mathrm{dec}}$ and $b_{\mathrm{dec}}$ are the weights and biases of the decoder convolutional layers,
- $h_{\mathrm{inc}}$ is the encoded feature map,
- σ is the activation function.

**SkipCAE** is a Convolutional Autoencoder version that incorporates skip connections to improve feature propagation through the network. Unlike traditional CAEs that rely entirely on layered encoding and decoding, SkipCAE integrates direct connections between corresponding layers of the encoder and decoder. These skip connections help maintain the spatial resolution of the image throughout the compression and reconstruction process, effectively mitigating the loss of detail that often occurs in deep architectures. By allowing information to bypass some of the convolutional and pooling layers, SkipCAE ensures that important features are not lost and can be effectively used for reconstruction [28].

Mathematically, the encoder and decoder processes for SkipCAE are described as follows:

Encoder:

$$h_{1}=\sigma \left(W_{1}*x+b_{1}\right),\;h_{2}=\sigma \left(W_{2}*h_{1}+b_{2}\right),\;\ldots$$

where

- *x* is the input image.
- $W_{1},W_{2},\ldots$ are the weights of the filters for each layer.
- $b_{1},b_{2},\ldots$ are the biases corresponding to each filter.
- ∗ represents the convolution operation.
- σ is the activation function (e.g., ReLU or LeakyReLU).
- $h_{1},h_{2},\ldots$ represent intermediate feature maps at different stages of the encoder.

Decoder: The decoder reconstructs the image from the encoded features, utilizing skip connections to add earlier feature maps to the decoding process:

$$x^{\prime }=\sigma \left(W_{\mathrm{dec}}*h_{2}+b_{\mathrm{dec}}\right)+\mathrm{skip}\left(h_{1}\right)$$

where

- $x^{\prime }$ is the reconstructed output image.
- $W_{\mathrm{dec}}$ and $b_{\mathrm{dec}}$ are the weights and biases of the decoder convolutional layers.
- $h_{2}$ is the encoded feature map from the encoder.
- $\mathrm{skip}(h_{1})$ denotes the feature map $h_{1}$ being added to the decoder at the corresponding layer to preserve finer details from the original image.
- σ is the activation function (e.g., ReLU or LeakyReLU).

**ResNetCAE**, incorporates the structure of a convolutional autoencoding (CAE) network with the deep learning capabilities of residual networks (ResNets) [29]. ResNets are known for their ability to mitigate the vanishing gradient problem by introducing residual connections that skip one or more layers, allowing the model to learn more complex representations efficiently. In the context of ResNetCAE, these residual connections are used within the encoder to propagate gradients across layers, enabling faster training and improved performance. This architecture maintains the core functionality of standard CAEs (compression and reconstruction) while taking advantage of residual connections.

Mathematically, the encoder and decoder processes for ResNetCAE are described as follows:

Encoder: The encoder applies convolutional layers with residual connections. The residual connection adds the input directly to the output of the convolutional layer to facilitate the learning process:

$$h=\sigma (W_{\mathrm{enc}}*x+b_{\mathrm{enc}})+x$$

where

- *x* is the input image.
- $W_{\mathrm{enc}}$ and $b_{\mathrm{enc}}$ are the weights and biases of the encoder convolutional layers.
- ∗ represents the convolution operation.
- σ is the activation function (e.g., ReLU).
- *h* is the feature map produced by the encoder.
- The addition of *x* represents the residual connection that helps the network learn the identity function, making the training process easier.

Decoder: The decoder reconstructs the image from the encoded features by applying convolutional layers

$$x^{\prime }=\sigma \left(W_{\mathrm{dec}}*h+b_{\mathrm{dec}}\right)$$

where

- $x^{\prime }$ is the reconstructed output image.
- $W_{\mathrm{dec}}$ and $b_{\mathrm{dec}}$ are the weights and biases of the decoder convolutional layers.
- *h* is the encoded feature map from the encoder.
- σ is the activation function (e.g., ReLU).

**MVTecCAE** is designed for the analysis of industrial quality inspection images, particularly in the context of MVTec datasets [30]. MVTecCAE incorporates a modular architecture that combines convolutional layers with attention mechanisms to focus on specific areas of the image that are more likely to contain defects. This allows the model to pay more attention to regions with potential problems such as scratches, stains or distortions. It also incorporates strategies for dealing with high-dimensional data and noise. Mathematically, the encoder and decoder processes for MVTecCAE are described as follows:

Encoder: The encoder uses multiple layers of convolutional operations, gradually reducing the spatial dimensions of the input image while increasing the depth of feature maps:

$$h_{1}=\sigma \left(W_{1}*x+b_{1}\right)\;h_{2}=\sigma \left(W_{2}*h_{1}+b_{2}\right)\;\vdots \;h_{n}=\sigma \left(W_{n}*h_{n-1}+b_{n}\right)$$

where

- *x* is the input image,
- $W_{1},W_{2},\ldots ,W_{n}$ are the weights of the convolutional layers,
- $b_{1},b_{2},\ldots ,b_{n}$ are the biases corresponding to each convolutional layer,
- ∗ represents the convolution operation,
- σ is the activation function (e.g., ReLU),
- $h_{1},h_{2},\ldots ,h_{n}$ are the feature maps produced at each layer.

Bottleneck Layer: After multiple convolutional layers, the feature maps are reduced to a compact representation:

$$h_{\mathrm{bottleneck}}=\sigma \left(W_{\mathrm{bottleneck}}*h_{n}+b_{\mathrm{bottleneck}}\right)$$

where

- $h_{\mathrm{bottleneck}}$ represents the compact representation of the input image,
- $W_{\mathrm{bottleneck}}$ and $b_{\mathrm{bottleneck}}$ are the weights and biases of the bottleneck layer,
- ∗ represents the convolution operation.

Decoder: The decoder attempts to reconstruct the original image from the compressed feature map by gradually increasing the spatial dimensions using deconvolutional (upsampling) layers:

$$x^{\prime }=\sigma \left(W_{\mathrm{dec}}*h_{\mathrm{bottleneck}}+b_{\mathrm{dec}}\right)$$

where

- $x^{\prime }$ is the reconstructed output image,
- $W_{\mathrm{dec}}$ and $b_{\mathrm{dec}}$ are the weights and biases of the decoder convolutional layers,
- $h_{\mathrm{bottleneck}}$ is the compressed feature map,
- σ is the activation function (e.g., ReLU).

## 5. Experiments and Results

In this section, we will describe the experiments that were performed and the results of those experiments.

### 5.1. Experiments Setup

#### 5.1.1. Models Setup

**BaselineCAE**: For the implementation of this model we employed an architecture as similar as possible to the original baseline CAE [26]. For this, we developed a created pipeline for training and evaluation. The encoder progressively reduces the input dimensions through layers of convolution, max-pooling, and activation functions, finishing in a dense bottleneck layer that captures the essential features of the input. The decoder then mirrors this structure, reconstructing the input image from the compact representation. During training, the model minimizes the mean squared error (MSE) between the input and reconstructed images.

**InceptionCAE**: For this model we employed an adaptation of the Inception model to work as a convolutional autoencoder [31]. This architecture integrates Inception-like layers, inspired by the original Inception module [27], to combine convolutional filters of multiple kernel sizes (1 × 1, 3 × 3, and 5 × 5) alongside a max-pooling operation. Each convolutional operation is followed by Batch Normalization and a Leaky ReLU activation, except for the final layer, which uses a Sigmoid activation. The encoder progressively reduces the spatial dimensions through three Inception modules, coupled with max-pooling layers, resulting in a bottleneck layer. The decoder mirrors this structure, employing upsampling layers to reconstruct the input.

To extract the low-dimensional representation of images, we applied a Global Average Pooling (GAP) operation to the bottleneck layer. The GAP operation computes the average value across spatial dimensions. This approach allows for a wider bottleneck layer compared to the BaselineCAE architecture, enhancing the representation capacity of the model. The InceptionCAE is trained on normal images using a mean squared error loss to minimize the reconstruction error between the input and output.

**SkipCAE**: For this model we implemented a model that uses skip connections [28]. In this model the encoder begins with a series of convolutional layers, each employing 3 × 3 filters and LeakyReLU activations, progressively downsampling the input image to extract important features. At specific stages, intermediate feature maps are preserved as “skip connections”, which capture spatial and contextual information from early encoding layers. These skip connections are later reintroduced in the decoder, where transposed convolutional layers gradually upsample the compressed latent representation back to the original image dimensions. The decoder integrates these preserved features using element-wise addition, ensuring that fine-grained details lost during downsampling are reinstated.

**ResNetCAE**: For this we used a ResNet-18 architecture as the backbone for the encoder. This encoder leverages ResNet’s residual blocks to extract deep-level features, resulting in two successive 512-filter convolutional layers that form the compressed latent representation. The decoder mirrors the complexity of the encoder with a series of transposed convolutional layers mixed with batch normalization and ReLU activations to ensure stable gradients and efficient upsampling. Residual links are a characteristic of this architecture; outputs from each upsampling layer are added to subsequent layers on an element-by-element basis, reinforcing feature propagation and mitigating information loss during decoding. This use of residual connections in the decoder not only complements the ResNet-based encoder but also significantly improves reconstruction accuracy, allowing the model to produce highly detailed outputs.

**MVTecCAE**: For this one we employ the same structure as the one proposed in [30]. This architecture, like the others, consists of an encoder and a decoder. In this case, the encoder starts with a series of convolutional layers that use kernels of multiple sizes, coupled with ReLU activation functions. The decoder mirrors this structure, using transposed convolutions and upsampling layers to reconstruct the input.

#### 5.1.2. Evaluation Metrics

In this study, to evaluate our results we will be focusing on two specific metrics. The first metric we will be using is the mean square error (MSE), which measures the average squared difference between the predicted and ground truth values. Mathematically, the MSE is defined as seen in Equation (1)

(1) $$MSE\left(x,y\right)=\frac{1}{n}\sum_{i=1}^{n}{\left(x_{i}-y_{i}\right)}^{2}\;$$

where $x_{i}$ represents the true pixel value and $y_{i}$ the reconstructed pixel value from the model, and n is the total number of pixels in the image. Lower MSE values indicate a better reconstruction performance, as they reflect smaller discrepancies between the original and reconstructed images. This metric is particularly sensitive to large errors due to the squaring operation, making it effective for identifying models that fail to accurately reproduce key image features.

The second evaluation metric that we will be using is the Structural Similarity Index Measure (SSIM), which evaluates the perceived quality of reconstructed images by comparing structural, luminance, and contrast information between the original and reconstructed versions. SSIM operates under the assumption that structural information is the primary factor in human visual perception. It computes subcomponents related to structure, luminance, and contrast changes, where the structure is obtained from autocorrelations and cross-correlations between neighboring pixels in the images. Normalization in these components accounts for the human visual system’s higher sensitivity to distortions in darker and lower contrast regions [32]. The formula used to calculate this metric is presented in Equation (2)

(2) $$SSIM\left(x,y\right)=\frac{\left(2\mu_{x}\mu_{y}+C_{1}\right)+\left(2\sigma_{xy}+C_{2}\right)}{\left(\mu_{x}^{2}+\mu_{y}^{2}+C_{1})(\sigma_{x}^{2}+\sigma_{y}^{2}+C_{2}\right)}$$

where $\mu_{x}$ and $\mu_{y}$ are the mean intensities of the original and reconstructed images, $\sigma_{x}^{2}$ and $\sigma_{y}^{2}$ are their variances, $\sigma_{xy}$ is their covariance, and $C_{1}$ and $C_{2}$ are small constants to stabilize the division. SSIM produces values in the range [−1, 1], where a value of 1 indicates perfect structural similarity.

Mean Square Error (MSE) and Structural Similarity Index Measure (SSIM) are both widely used metrics for evaluating image reconstruction quality, but they are quite different in their approach and focus. MSE is a pixel-wise error metric very sensitive to large errors and SSIM is designed to more closely mimic the human visual system by focusing on structural information, luminance, and contrast. While MSE provides a straightforward measure of numerical accuracy, SSIM provides a perceptually meaningful evaluation, making it more appropriate for tasks where visual quality is critical. Together, these metrics provide complementary insights into both the quantitative accuracy and perceptual fidelity of reconstructed images.

### 5.2. Results and Analysis

Twenty different models were trained, corresponding to the five CAE architectures combined with the four different piece types. To ensure consistent and reliable training across all models, each one was trained for 300 epochs using a fixed learning rate of 0.001, the Adam optimizer, and the mean square error (MSE) as the loss function.

As mentioned previously we used four different piece types, two simple sub-assemblies and two minor sub-assemblies. In this section, we will refer to the simple sub-assemblies as pieces A and D, and the minor sub-assemblies as pieces B and C.

**Figure 7:**
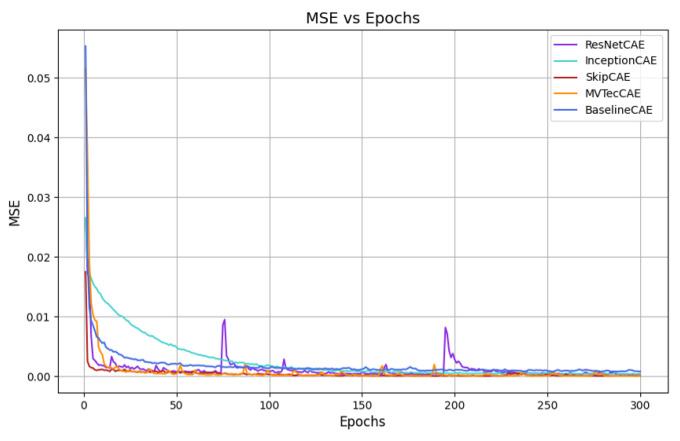
MSE evolution over epochs for Piece A (validation).

**Figure 8:**
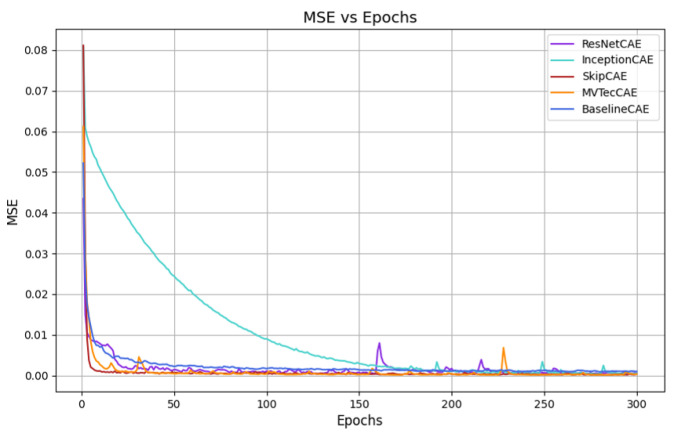
MSE evolution over epochs for Piece B (validation).

**Figure 9:**
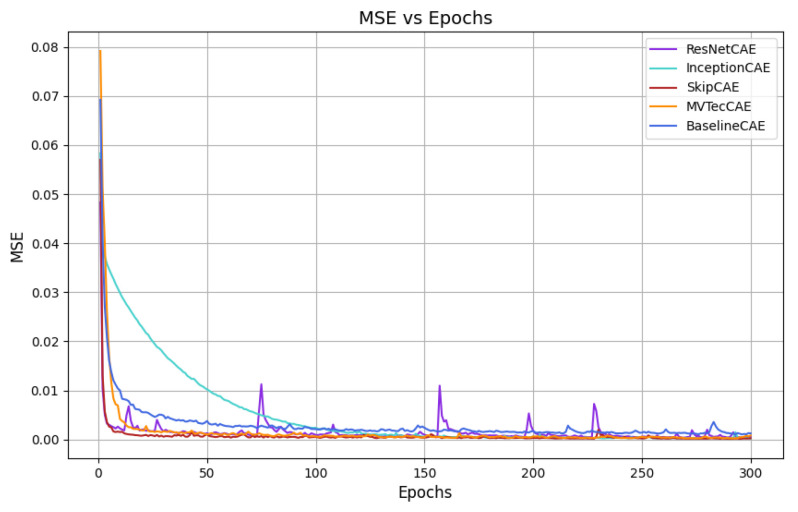
MSE evolution over epochs for Piece C (validation).

**Figure 10:**
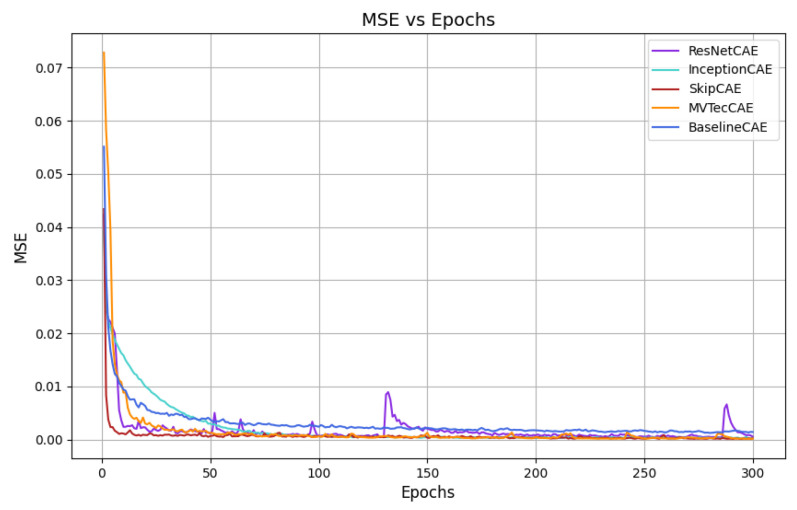
MSE evolution over epochs for Piece D (validation).

Figure 7, Figure 8, Figure 9 and Figure 10 provide the graphical representation of the mean squared error (MSE) values over the epochs during the validation process for each piece type. In these figures, each color corresponds to a different CAE model, allowing for a clear visual comparison of the performance of each approach across the different piece types.

These plots illustrate how the MSE values of the models evolve over time, helping to highlight their learning behavior, stability, and effectiveness for each piece. The decrease in MSE is evident for all models, with some CAEs, such as InceptionCAE, showing a slower decrease in the MSE, while others, such as SkipCAE, show a faster decrease. Despite these differences in the rate of convergence, all models achieve low MSE values at the end of the 300 epochs, demonstrating their ability to effectively learn and reconstruct the pieces over time.

After training, the next step was to determine the threshold for distinguishing non-defective images from defective ones. This threshold was selected by calculating the MSE for each data element in the training set and identifying the highest observed MSE value. This maximum MSE value was then used as the threshold.

**Figure 11:**
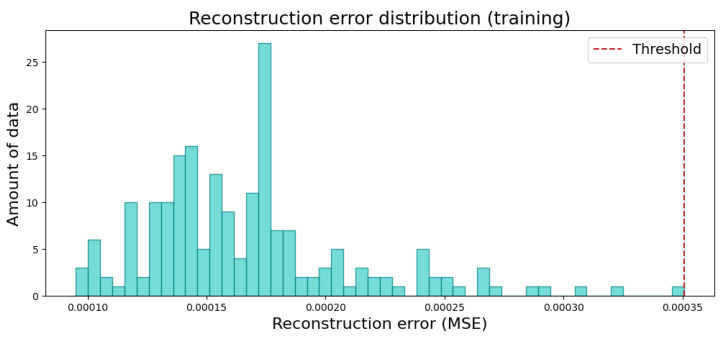
An example of the MSE error for the training dataset and the threshold selected.

A more visual representation of this process is shown in Figure 11, where the MSE values for all elements in the training set are plotted along with the selected threshold corresponding to the highest MSE value. In this case, the model used was MVTecCAE and the data from piece D.

The threshold selection process was conducted for all twenty trained models. The resulting threshold values for each model and piece type are shown in Table 1. From this table, we can see that the lowest MSE values were obtained for the simple sub-assemblies (pieces A and D) and for one of the minor sub-assemblies when using the MVTecCAE model. For the other piece, the lowest MSE value was obtained using InceptionCAE.

Once the thresholds were set, we proceeded to test the models. For testing, we used 20% of the images from the non-defective parts, along with a set of images containing defective parts. This mixed dataset allowed us to evaluate the model’s ability to accurately distinguish between non-defective and defective parts.

**Figure 12:**
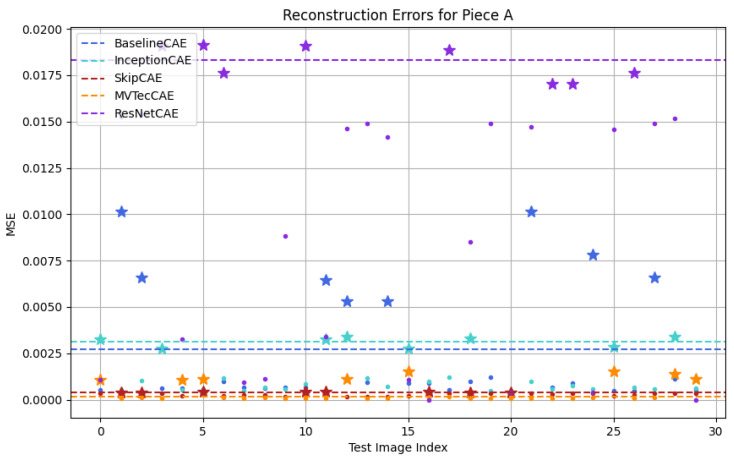
Testing results for Figure A (Stars: Defective Pieces, Dots: Non-Defective Pieces, Dash Lines: CAE Thresholds, Colors: Different CAEs).

**Figure 13:**
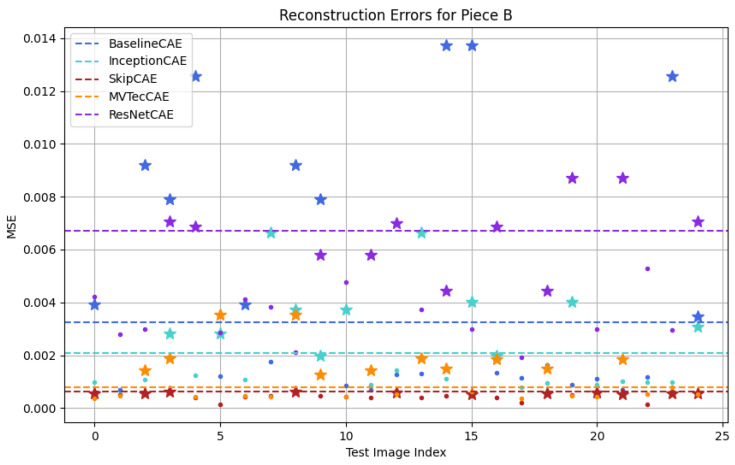
Testing results for Figure B (Stars: Defective Pieces, Dots: Non-Defective Pieces, Dash Lines: CAE Thresholds, Colors: Different CAEs).

**Figure 14:**
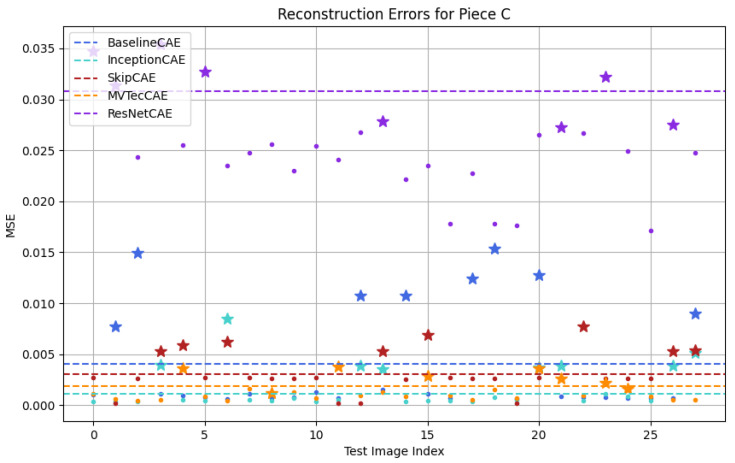
Testing results for Figure C (Stars: Defective Pieces, Dots: Non-Defective Pieces, Dash Lines: CAE Thresholds, Colors: Different CAEs).

**Figure 15:**
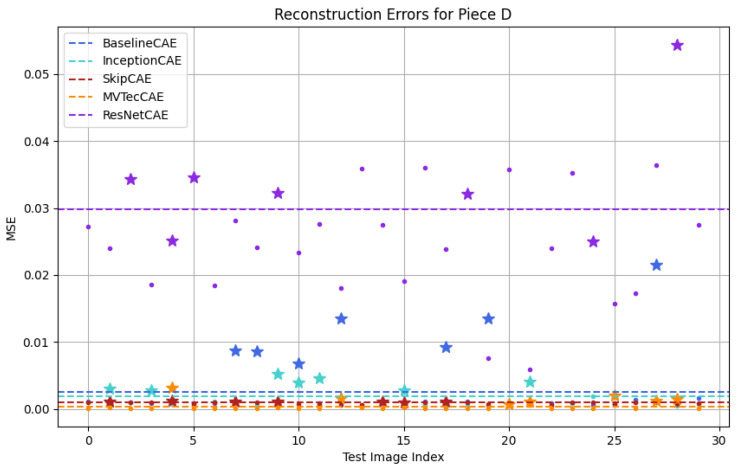
Testing results for Figure D (Stars: Defective Pieces, Dots: Non-Defective Pieces, Dash Lines: CAE Thresholds, Colors: Different CAEs).

Figure 12, Figure 13, Figure 14 and Figure 15 show a graphical representation of the results obtained from the test dataset for each piece type. In these plots, each color corresponds to a different CAE model, with the threshold indicated by a dashed line. As observed, each CAE produces different MSE values for each image, reflecting the different performance across all models.

To make it easier for us to graphically identify the defective pieces, we have represented the defective pieces with stars and the non-defective pieces with dots. To evaluate whether the model correctly identifies anomalies, we must focus on the threshold. If a dot or star is below the threshold line, it indicates that the piece is classified as non-defective, whereas if it is above the threshold, the piece is classified as defective. This distinction allows us to evaluate how accurately the model detects defects in the test dataset.

Figure 12, Figure 13, Figure 14 and Figure 15 provide a graphical representation of the results, with each color corresponding to a different CAE. The dashed lines indicate the respective thresholds for each CAE, corresponding to their assigned colors. Defective pieces are represented by stars, while non-defective pieces are represented by dots, both color-coded according to the CAE used.

For piece A, as shown in Figure 12, the BaselineCAE and MVTecCAE successfully classified all defective images and distinguished them from non-defective images. InceptionCAE was able to identify 62.5% of the defective pieces. In contrast, the SkipCAE and ResNetCAE performed significantly worse, correctly identifying only 50% of the defective pieces.

In the case of piece B (Figure 13), the BaselineCAE and MVTecCAE achieved perfect detection, accurately distinguishing all defective images from non-defective ones. In comparison, the InceptionCAE identified 81.82% of the defective pieces, while the ResNetCAE and SkipCAE identified 63.64% and 45.46%, respectively.

For piece C, as shown in Figure 14, the BaselineCAE, InceptionCAE and SkipCAE showed flawless detection, successfully distinguishing all defective images. However, the MVTecCAE identified 75% of the defective pieces and the ResNetCAE identified 62.5%.

Finally, for Piece D (Figure 15) the BaselineCAE, InceptionCAE, and MVTecCAE were able to correctly classify all defective images. The ResNetCAE identified 71.43% of the defective pieces, while the SkipCAE identified 57.14%

**Figure 16:**
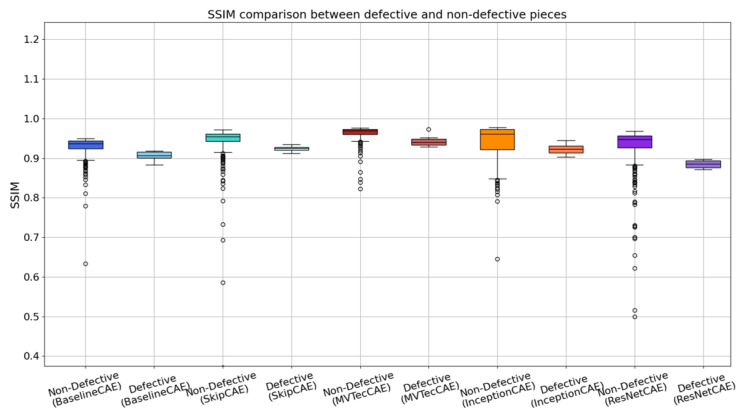
Boxplot of the results of each CAE for the test and train data.

Figure 16 shows a box plot illustrating the SSIM comparison between defective and non-defective pieces of type C for each of the five CAE models. Unlike MSE, which directly measures the reconstruction error, SSIM evaluates the perceptual similarity between the original and reconstructed images by taking into account structural, luminance, and contrast information. While MSE provides a clear indication of how well the model can reconstruct an image, it may not reflect the visual quality or subtle differences in texture and structure that are important for defect detection. On the other hand, SSIM provides a more complete view by capturing these perceptual aspects, making it a valuable metric for confirming the results obtained from MSE. Each boxplot shows the median SSIM values, inter-quartile ranges, and potential outliers, allowing us to compare the structural similarities of the reconstructed images.

In the box plot (Figure 16), it is visible that for each CAE model, the SSIM values for the non-defective pieces are consistently higher compared to the defective pieces. The non-defective pieces tend to have higher median SSIM values, indicating that the models can more accurately reconstruct these images with better structural and perceptual similarity to the original. In contrast, the defective pieces have lower SSIM scores, reflecting the inability of the model to accurately reconstruct the images due to the overshooting defects, resulting in structural distortions. The box plot also highlights the variability in the SSIM values for the defective parts, which tend to have a wider range, suggesting that the severity of the defects has a greater impact on the model’s reconstruction quality.

**Figure 17:**
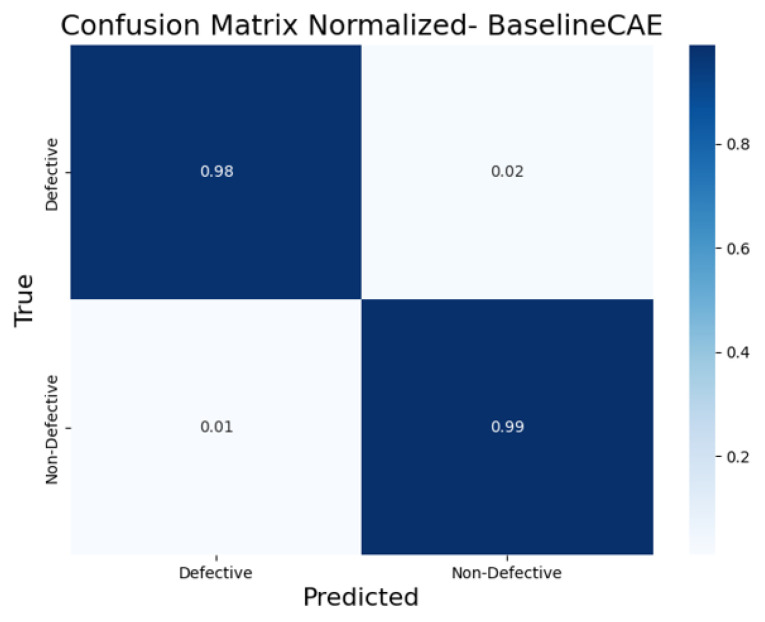
Confusion matrix normalized for BaselineCAE).

**Figure 18:**
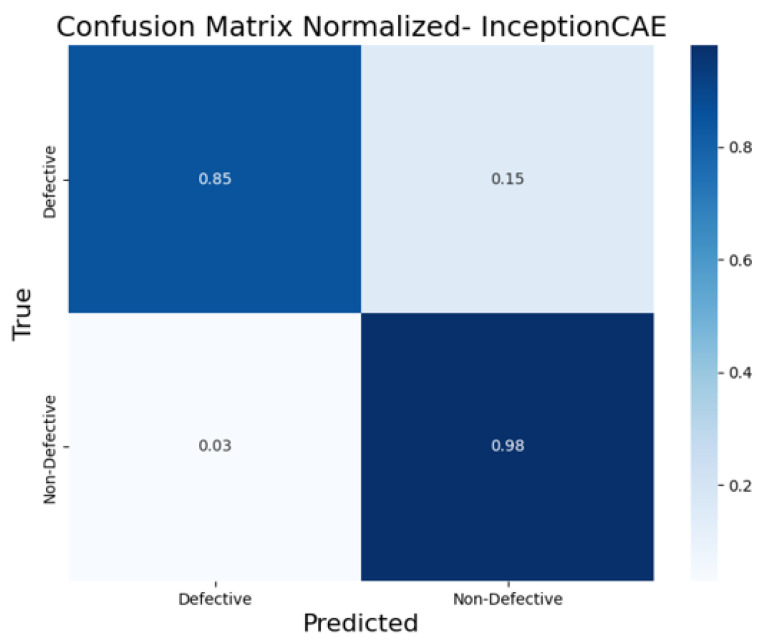
Confusion matrix normalized for InceptionCAE.

**Figure 19:**
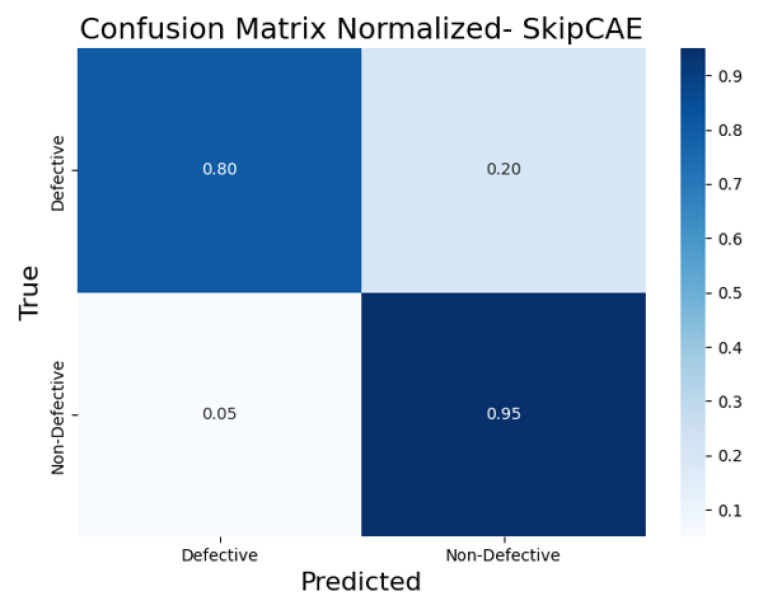
Confusion matrix normalized for SkipCAE.

**Figure 20:**
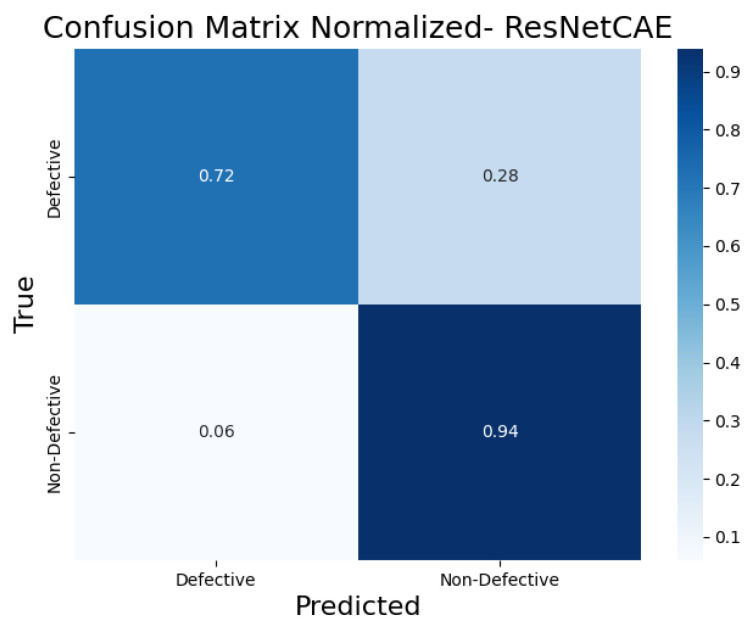
Confusion matrix normalized for ResNetCAE.

**Figure 21:**
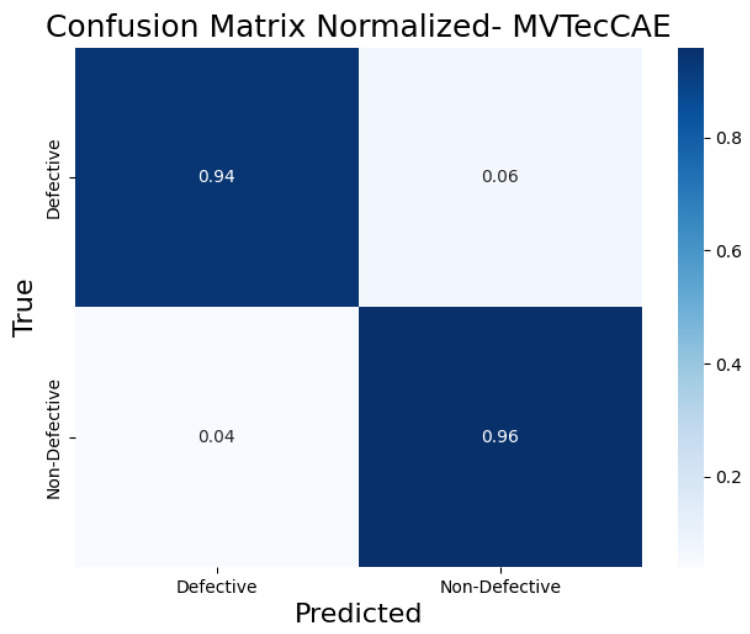
Confusion matrix normalized for MVTecCAE.

To further evaluate the classification performance of each CAE model, the normalized confusion matrices (Figure 17, Figure 18, Figure 19, Figure 20 and Figure 21) are shown, where the results for each piece have been grouped to simplify the analysis. These matrices provide a global view of the proportion of correctly and incorrectly classified pieces, providing a deeper insight into each model’s ability to distinguish defective from non-defective pieces. By normalizing the confusion matrices, we enable a fair comparison between the different CAEs.

The BaselineCAE (Figure 17) performed exceptionally well, with a high true positive rate for defective pieces (98%) and a very low false positive rate for non-defective pieces (only 2%). It also correctly classified 99% of the non-defective pieces. This indicates that the BaselineCAE is very reliable in both identifying defects and distinguishing non-defective items, leading to highly accurate classifications across both defective and non-defective samples.

The InceptionCAE (Figure 18) displayed good performance, identifying 85% of the defective pieces correctly. However, it had a higher false positive rate (15%) compared to the BaselineCAE, indicating some confusion between defective and non-defective pieces. The model also correctly classified 98% of the non-defective pieces.

The SkipCAE (Figure 19) model achieved an 80% true positive rate for defective pieces but with a 20% false positive rate. While it showed reasonable accuracy in detecting defective items, its higher false positive rate suggests it occasionally misclassifies non-defective pieces as defective. The non-defective classification accuracy was also lower at 95%.

The ResNetCAE (Figure 20) had the lowest true positive rate for defective pieces at 72%, and a 28% false positive rate. This indicates that while it can identify some defective pieces, it has more difficulty than the other models, and misclassifies a significant number of non-defective pieces, with only 94% of the non-defective pieces being correctly classified.

The MVTecCAE (Figure 21) performed very well, correctly identifying 94% of the defective pieces and having a low false positive rate of only 6%. It also classified 96% of the non-defective pieces correctly. These results indicate that the MVTecCAE is highly effective in distinguishing between defective and non-defective pieces with a good balance between sensitivity and specificity.

Table 2 summarizes the computational aspects of the five CAE models, providing insights into their training time, number of parameters, and GPU VRAM usage. The average training time represents the time required for each model to complete training, with the SkipCAE being the fastest, requiring 893.79 s, while the ResNetCAE takes significantly longer at 8529.74 s. This difference in training time can be attributed to the complexity of the models, with ResNetCAE having the highest number of trainable and non-trainable parameters. The number of trainable parameters indicates the number of parameters learned by the model during training. ResNetCAE has the highest number of trainable parameters with 34,935,427, reflecting its deeper and more complex architecture, while MVTecCAE has the lowest with only 751,332 trainable parameters. InceptionCAE and SkipCAE fall in between, with InceptionCAE requiring a significant number of parameters (11,519,241) while SkipCAE uses 3,057,635 trainable parameters. The non-trainable parameters are those that are fixed and do not change during training. These are generally associated with aspects such as pre-trained layers or architectural choices that do not require training. ResNetCAE has the most non-trainable parameters (56,960), while MVTecCAE has no non-trainable parameters, suggesting a simpler model architecture. Finally, the computational load is indicated by the GPU VRAM used during training. ResNetCAE requires the most memory (7.9 GB), while SkipCAE requires the least (5.1 GB). This further highlights the inherent relationship between model complexity and resource consumption, with more complex models requiring more computational resources.

To summarize the key aspects and limitations of the work, we provide Table 3. This table highlights the specifications and limitations associated with various components of the study. Each row focuses on an important aspect, such as the model architectures used, the dataset employed, computational resources, and generalizability. It also includes considerations specific to the industrial context, acknowledging both the strengths and limitations of the methods and data used in this work.

## 6. Conclusions and Future Works

Convolutional Autoencoders (CAEs) are widely used in diverse fields, especially in unsupervised learning tasks such as anomaly detection. Their ability to learn optimal representations of input data through compression and subsequent reconstruction makes them highly effective for detecting deviations from normal patterns.

In the context of defect detection, CAE’s are particularly useful because they can detect subtle changes in data, such as structural anomalies or defects, that may not be immediately apparent. This makes them an excellent tool for quality control processes, where ensuring that products meet specific standards is very important.

The results obtained in this study showed significant performance differences among the evaluated CAEs, with MVTecCAE consistently achieving the lowest MSE scores across most of the piece types. The only exception was observed in one simple sub-assembly, where InceptionCAE outperformed MVTecCAE. To validate these findings, we also used the Structural Similarity Index Measure (SSIM), a metric that evaluates the perceptual quality of the reconstructed images. By analyzing the SSIM values for both defective and non-defective pieces, we found that MVTecCAE and InceptionCAE exhibited the highest SSIM values for non-defective pieces, indicating their ability to accurately reconstruct images of high quality. However, when the models were presented with defective pieces, SSIM values decreased, confirming that both CAEs successfully detected anomalies through reduced structural similarity. This reduction in SSIM and increase in MSE score for defective pieces is a strong indicator that the models are distinguishing between normal and defective parts, further supporting the effectiveness of CAEs in automated defect detection.

As for the rest of the CAEs, we observe that ResNetCAE consistently achieved the highest Mean Squared Error (MSE) values, indicating its poorer performance compared to the other CAEs. It was followed by SkipCAE, which also produced relatively high MSE values, suggesting a moderate level of performance in defect detection. On the other hand, BaselineCAE and InceptionCAE exhibited similar MSE results, with InceptionCAE slightly outperforming BaselineCAE in most cases, demonstrating its relatively better ability to identify defects. However, as said before, the standout model was MVTecCAE. This clear ranking suggests that MVTecCAE, InceptionCAE and BaselineCAE are the most effective models for detecting defects in this specific context, while ResNetCAE and SkipCAE are less effective in this specific application.

Regarding the impact of the camera used in this work, the proposed methodology is adaptable to other cameras, resolutions, and imaging conditions. In addition, our dataset was designed to include a wide range of variations, including different distances, lighting conditions, and angles, reflecting the dynamic environment of an industrial setting. This variation was intentionally included to ensure that our models could handle a variety of real-world conditions, thus ensuring generalization to different imaging setups.

By evaluating these CAE models, we were able to assess their effectiveness in reconstructing non-defective pieces and identifying defects based on the differences in reconstruction quality. This approach underscores the importance of using CAEs in the context of quality control. By implementing CAEs for defect detection, companies can automate the detection process, ensuring that each and every product is free from defects.

For future work, we first suggest expanding the dataset used to train and test the models. Currently, the study focused on a limited number of simple and small subassemblies, but expanding to a more diverse set of pieces, including more complex subassemblies, could help increase the robustness and generalizability of the model. Additionally, integrating different types of data, such as sensor data or 3D scans, could improve the detection capabilities and provide a more comprehensive analysis of defects. Another possible future work is to refine the threshold selection process for classifying defective and non-defective parts. Developing more sophisticated thresholding techniques, such as adaptive thresholding based on dynamic learning, could improve the accuracy of defect detection and reduce false positives or negatives. Also, we suggest exploring the effect of including both defective and defect-free samples in the training process, this analysis could help evaluate how the model performs when it is exposed to defects during training. Finally, real-time defect detection systems could be developed to enable the real-time evaluation of components as they are manufactured. Implementing such systems would integrate CAEs into the quality control workflow, allowing for immediate feedback and faster response times in identifying and addressing defects.

## Figures and Tables

**Table 1:** MSE threshold for each piece and each convolutional autoencoder.

	BaselineCAE	InceptionCAE	SkipCAE	ResNetCAE	MVTecCAE
Piece A	0.0027	0.0031	0.0004	0.0183	0.0002
Piece B	0.0032	0.0021	0.0061	0.0067	0.0008
Piece C	0.0041	0.0011	0.0031	0.0308	0.0019
Piece D	0.0026	0.0018	0.0011	0.0298	0.0004

**Table 2:** Performance of different CAE models based on training time, parameters, and computational load.

	Average Training Time	Trainable Parameters	Non-Trainable Parameters	Computational Load (GPU VRAM)
BaselineCAE	922.45 s	2,496,931	800	5.3 GB
InceptionCAE	1684.09 s	11,519,241	7174	6.2 GB
SkipCAE	893.79	3,057,635	384	5.1 GB
ResNetCAE	8529.74	34,935,427	56,960	7.9 GB
MVTecCAE	3616.23 s	751,332	0	5.4 GB

**Table 3:** Specifications and limitations of the work.

Aspect	Specification	Limitation
Dataset	Dataset consists of 4 piece types: 2 simple sub-assemblies and 2 minor sub-assemblies.	Dataset limited to these 4 types
Computational Resources	Training conducted on Nvidia RTX 4070 Ti	Training with other GPUs with less powerful hardware could affect training time
Generalizability	Results validated on the selected dataset.	Different dataset were not tested
Industrial Context	Data augmentation techniques were used to simulate a range of scenarios.	Industrial environments are complex and dynamic, there could be unforeseen scenarios.

## Data Availability

The data presented in this study are available on request from the corresponding author.
